# Truss structure optimization via hierarchical tree search

**DOI:** 10.1186/s40323-026-00320-1

**Published:** 2026-05-12

**Authors:** Arvan Sedighzadeh, Matteo Torzoni, Alberto Corigliano

**Affiliations:** Department of Civil and Environmental Engineering, Politecnico di Milano, Milan, Italy

**Keywords:** Truss optimization, Hierarchical Monte Carlo tree search, Reinforcement learning, Structural adaptivity

## Abstract

Truss design is a highly constrained problem due to mechanical requirements and practical limitations related to fabrication and assembly. This study formulates truss design synthesis as a discrete Markov decision process, in which grammar-constrained actions generate feasible intermediate layouts and Monte Carlo Tree Search (MCTS) learns an optimal design policy. Previous work has shown that MCTS outperforms both metaheuristic methods, such as genetic algorithms, and alternative reinforcement learning approaches, including Q-learning and deep Q-learning. However, its computational scalability is limited by the rapid growth of admissible configurations in dense grid design domains. To address these limitations, we propose a Hierarchical MCTS (H-MCTS) framework in which staged grid refinements focus computational resources on promising regions of the domain, thereby alleviating the curse of dimensionality. Benchmark evaluations show that H-MCTS consistently improves design quality and reduces computational cost compared to single-stage MCTS. To accommodate variable design conditions, H-MCTS is further applied to on-the-fly structural adaptivity through an offline–online strategy that precomputes optimal solutions and interpolates them in real time. The effectiveness of the computational procedure is demonstrated on a bridge-like truss structure that is progressively constructed and then morphed to accommodate moving loads and localized damage.

## Introduction

Computational design synthesis is a framework for automating structural design, supported by physics-based simulation and algorithmic decision-making [1]. Within this context, truss optimization has been extensively investigated owing to its relevance to a wide range of engineering applications, including lightweight structures and deployable systems. However, while continuum approaches such as the solid isotropic material with penalization method [2–9] and level-set formulations [10–15] have demonstrated effective for topology optimization, these methods can not directly operate on discrete members. The generated layouts often require substantial post-processing to obtain manufacturable truss configurations [2, 16].

Bio-inspired and metaheuristic algorithms such as genetic algorithms, particle swarm optimization, simulated annealing, and ant colony optimization have been widely used to address size, shape, and topology optimization [17–20]. However, their reliance on population-based evolution and stochastic sampling often results in slow convergence, sensitivity to parameter tuning, premature stagnation, and a heavy dependence on penalty functions to ensure stability and stress admissibility [21–23]. In addition, their high computational cost limits their practical applicability to real-world problems [18, 23]. These constraints have motivated a shift toward learning-based strategies for sequential design in the presence of delayed rewards.

Reinforcement Learning (RL) has emerged as a promising approach for modeling sequential design actions. Early contributions have employed image-based and graph-based RL formulations to generate or prune truss layouts [24–26]. More recently, Ororbia and Warn have formalized truss optimization as a Markov Decision Process (MDP), adopting generative grammar rules to ensure admissible intermediate designs prior to reaching the final structure [27]. Their Q-learning implementation enables reproducing globally optimal topologies on small benchmarks. However, such tabular RL methods scale poorly as the state–action space grows, which has motivated Ororbia and Warn to develop a Deep Reinforcement Learning (DRL) framework [28], where tabular value storage is replaced with neural function approximators. While DRL improves scalability and solution quality, these methods suffer from poor reward backpropagation in delayed-reward settings typical of structural optimization [29], instability in neural approximators, extensive training demands, and a lack of interpretability in the learned policies [30].

Among RL techniques, tree-search methods have gained traction in truss optimization due to their balanced exploration–exploitation behavior and their sample-efficient use of simulated experience [31, 32]. Specifically, Monte Carlo Tree Search (MCTS) has been applied to discrete design in frameworks such as AlphaTruss [33] and KR-UCT—the latter being an Upper Confidence Bounds for Trees (UCT) method combined with Kernel Regression (KR) [34]—demonstrating superior performance compared to metaheuristics and other RL approaches. Moreover, the MCTS formulation of Garayalde et al. [29] has demonstrated that grammar-guided tree search can reduce the number of Finite Element (FE) evaluations by more than half compared to DRL, while consistently achieving globally optimal or near-optimal trusses. Their results have further revealed that the backpropagation mechanism of MCTS is naturally suited to the delayed-reward structure of truss design, allowing information to be propagated from terminal to early decision nodes.

Despite these advances, three major challenges remain unaddressed in the existing literature.

First, scalability deteriorates as the number of nodes in the design domain increases. Dense environments expand the action branching factor exponentially, causing rapid degradation of computational performance for both DRL and tree-search methods [31, 33, 34]. Second, on-the-fly adaptivity to changing external conditions has not yet been fully integrated into topology synthesis. For instance, existing RL and MCTS formulations typically operate under fixed loading conditions. Although progressive construction has been explored in [29], no available approaches enable continuous morphing of the layout in response to evolving load configurations. Such a capability would be highly beneficial in engineering applications like bridge decks, crane booms, or adaptive mechanical systems. Third, damage awareness remains absent from current optimization frameworks. Structural degradation affects stiffness, stability, and admissible stress ranges, thereby influencing the optimal topology. Existing studies assume pristine conditions, leaving unanswered how optimal truss configurations should respond to localized damage and how damage regions should be reinforced to maintain adequate stiffness.

To address these gaps, we propose a Hierarchical Monte Carlo Tree Search (H-MCTS) framework. The method retains the grammar-based MCTS structure introduced in [29] but extends it through staged grid refinements. At each stage, a coarse MCTS identifies active, structurally relevant nodes, after which refinement zones are subsequently established around them. Higher-resolution grids are then restricted to these regions, allowing the algorithm to progressively reduce the dimensionality of the search space while preserving its structurally meaningful components. Building upon this hierarchical formulation, we further introduce an offline–online morphing strategy to manage moving loads and local damage. In the offline phase, H-MCTS is executed for several discrete load positions and potential damage scenarios, producing a database of optimized configurations that satisfy yield and buckling constraints. Online, new load positions and damage scenarios are handled by interpolating between stored topologies, while preventing member intersections and ensuring mechanical admissibility. Additional grammar-guided reinforcement is then applied in localized regions, enabling rapid reshaping without running full optimizations.

We present benchmark studies showing that H-MCTS consistently reduces both structural compliance and computational cost relative to standard MCTS, particularly in large domains where uniform-grid MCTS becomes computationally prohibitive. The hierarchical mechanism alleviates the curse of dimensionality and concentrates computational effort to structurally relevant regions. Moreover, we show that our offline–online strategy enables rapid morphing, allowing the structure to adapt in response to moving loads and the presence of degraded elements.

The remainder of the paper is structured as follows. Sect. “Optimal Truss Design via Constrained Tree Search” presents the methodological foundation, including the formulation of the optimal truss design problem, its MDP abstraction, and the grammar rules employed to guide the synthesis process. Sect. “Computational Framework” describes the proposed computational framework, beginning with the standard MCTS approach and then focusing on alternative selection policies, the hierarchical extension, and the offline–online morphing procedure. Sect. “Results” presents benchmark results, evaluates the performance of H-MCTS relative to standard MCTS, and examines a progressive construction setup that illustrates structural adaptation under moving loads and a representative damage scenario.

## Optimal truss design via constrained tree search

### Optimal design problem for truss structures

We formulate the design problem as finding a truss geometry that optimizes an objective function under static loading. In this study, the chosen objective is the minimization of the maximum absolute displacement, a formulation analogous to compliance minimization in topology optimization [35]. We employ a FE discretization and consider a planar truss structure composed of *I* truss elements. The optimization problem can thus be formulated as finding the set of truss elements $\left\{{\Omega }_{1}^{s},\dots , {\Omega }_{I}^{s}\right\}$ that minimizes the structure’s maximum displacement, as follows:

1 $$\underset{\Omega =\bigcup_{i=1}^{I}{\Omega }_{i}^{s}}{\mathrm{min}} {\Vert \mathbf{U}(\Omega )\Vert }_{\infty }, \text{with }{\Omega }_{i}^{s}\text{ a truss FE},$$

subject to:

2 $$\mathbf{K}\mathbf{U}=\mathbf{F}, \mathrm{in}\, \Omega =\bigcup_{i=1}^{I}{\Omega }_{i}^{s},$$

3 $$\mathbf{U}={\mathbf{U}}_{0},\text{ on }{\partial\Omega }_{g}=\bigcup_{i=1}^{I}{\partial\Omega }_{g i}^{s},$$

4 $$V\le {V}^{\mathrm{max}}, \text{with }V=\sum_{i=1}^{I}{A}_{i}{L}_{i},$$

where $\mathbf{U}$ is the vector of nodal displacements and ${\Vert \mathbf{U}(\Omega )\Vert }_{\boldsymbol{\infty }}$ denotes its infinity norm, defined as the maximum absolute value among its entries. This norm directly controls the worst-case nodal displacement and therefore the maximum deformation experienced anywhere in the structure, as commonly associated with serviceability requirements. Equations (2)–(4) represent the constraints over Problem (1), respectively: ensuring the linear elastic equilibrium under the nodal load vector $\mathbf{F}$ through the stiffness matrix $\mathbf{K}$; enforcing the prescribed displacement vector ${\mathbf{U}}_{0}$ on the Dirichlet boundary ${\partial\Omega }_{g}$; and limiting the total structural volume $V$-based on the cross-sectional areas ${A}_{i}$ and lengths ${L}_{i}$ of truss elements ${\Omega }_{i}^{s}$, $i=1,\dots ,I$-to a maximum allowed volume ${V}^{\mathrm{max}}$. Notably, this framework can be expanded to incorporate nonlinear constitutive behavior; for further details on the FE formulation, please refer to Ref. [36].

### Markov decision process for sequential decision-making

Markov decision processes provide a mathematical framework for sequential decision making. In MDPs, an agent sequentially interacts with an environment by taking actions that induce changes in the environment. The environment then returns the next state and assigns a reward associated with the executed action and the resulting configuration. The agent has the goal to learn a control policy—i.e. a mapping that selects actions based on the current state—that maximizes the expected cumulative reward over time. This framework is well suited to design synthesis problems, where each design modification affects not only the immediate structural response (immediate reward) but also the performance of all future configurations (delayed reward) [37].

Formally, an MDP is defined as a four-tuple $\langle \mathcal{S},\mathcal{A},\mathcal{P},\mathcal{R}\rangle .$ Here, $\mathcal{S}$ is the state space, representing all possible configurations the system can assume; $\mathcal{A}$ is the action space available to the agent; $\mathcal{P}$ is the Markov transition model, describing a probabilistic mapping that encodes the likelihood of moving from one state to another given a specific action; and $\mathcal{R}$ encodes a reward function, which assigns a numerical score to each state-action pair.

The planning horizon $(0,T)$ is discretized into nondimensional time steps $t=0,\dots ,T$. At each time $t$, the system occupies a state ${s}_{t}\in \mathcal{S}$, and the agent selects an action ${a}_{t}\in \mathcal{A}$. The transition model $\mathcal{P}:\mathcal{S}\times \mathcal{A}\times \mathcal{S}\to [\mathrm{0,1}]$ determines the probability of reaching any state ${s}_{t+1}$ at time $t +$ 1, given ${s}_{t}$ and ${a}_{t}$. The reward ${r}_{t}\in \mathcal{R}$ quantifies the utility of the state-action transition. The total performance over the planning horizon is typically expressed as the expected discounted cumulative reward.

The control policy $\pi :\mathcal{S}\to \mathcal{A}$ maps each state to an action. The objective is to identify the *optimal* control policy ${\pi }^{*}$, which yields the optimal action ${a}_{t}^{*}$ at every state ${s}_{t}$. This policy maximizes the total expected return, as quantified by the *action-value* function ${\mathcal{Q}}^{\pi }:\mathcal{S}\times \mathcal{A}\to \mathcal{R}$. Such function ${Q}^{\pi }\left({s}_{t},{a}_{t}\right)$ represents the expected cumulative reward obtained by taking action ${a}_{t}$ in state ${s}_{t}$, and subsequently following policy $\pi$.

For our truss design purposes, we employ a grid-world environment defined over a prescribed set of nodes. This discrete design domain determines the set of feasible topologies that can be generated from the admissible locations of truss nodes. While a different grid discretization may lead to variations in the resulting layouts, this behavior is typical of ground-structure approaches; as the grid resolution increases, the design space becomes progressively richer and the feasible solutions approach those of a continuous domain. The state space $\mathcal{S}$ includes any admissible truss layout that can be formed on this grid.

The action space $\mathcal{A}$ comprises any possible modification of a given layout. The reward function $\mathcal{R}$ may encode either local objectives, such as the displacement of a specified node, or global performance indicators, such as the maximum absolute displacement, stress levels, or strain energy. In this study, we use the maximum nodal displacement experienced by the structure, as:

5 $${r}_{t}=\left\{\begin{array}{c}0, if\,V>{V}^{\mathrm{max}} \,or\, {\Vert \mathbf{U}\Vert }_{\infty }>{\Vert {\mathbf{U}}_{\mathrm{init}}\Vert }_{\infty },\\ \frac{{\Vert {\mathbf{U}}_{\mathrm{init}}\Vert }_{\infty }-{\Vert \mathbf{U}\Vert }_{\infty }}{{\Vert {\mathbf{U}}_{\mathrm{init}}\Vert }_{\infty }}, \,otherwise.\end{array}\right.$$

where ${\Vert \mathbf{U}\Vert }_{\infty }$​ is the current maximum nodal displacement and ${\Vert {\mathbf{U}}_{\mathrm{init}}\Vert }_{\infty }$​ is that of the initial (seed) structure. This reward form ensures that ${r}_{t}\in [\mathrm{0,1}]$.

Modeling the design process as an MDP enables the use of RL techniques to address the complexity and dynamic nature of structural synthesis. However, the cardinality of both $\mathcal{S}$ and $\mathcal{A}$ grows rapidly even for small design domains. As a result, explicitly modeling the transition model $\mathcal{P}$ is impractical, if not impossible. Instead, optimal planning is achieved through simulated experience generated by the FE model in Eq. (2), which can be queried to produce a sample transition for any state–action pair. This setup is common in episodic RL, where environment trajectories are explored by repeatedly querying a simulator with control actions. Examples include Q-learning [27, 28] and MCTS [29], in which the action-value function is approximated and subsequently used as a proxy for the optimal control policy.

### Generative grammar rules for truss design synthesis

**Fig. 1:**
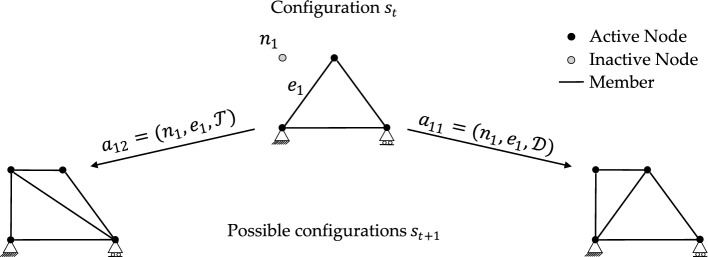
Representative actions under the $\mathcal{D}$ and $\mathcal{T}$ operators. From configuration ${s}_{t}$, action ${a}_{11}$ ($\mathcal{D}$) or ${a}_{12}$ ($\mathcal{T}$) updates the structure by pairing the selected element ${e}_{1}$ with the inactive node ${n}_{1}$

Grammar rules guide the generation of new truss configurations. The process begins from a seed configuration ${s}_{0}$, defined by deploying a few bars to create an admissible truss structure. This initial layout is then modified through a sequence of actions selected by the agent in compliance with the grammar. At each step, applying an allowed action to the current state ${s}_{\mathrm{t}}$ produces a new configuration ${s}_{t+1}$, as illustrated in Fig. 1. The process continues until a terminal state ${s}_{T}$ is reached, typically defined by a termination criterion such as attaining ${V}^{\mathrm{max}}$.

The grammar rules are employed to ensure that layout modifications respect mechanical principles, thereby keeping intermediate truss patterns mechanically admissible. Specifically, we enforce the formation of intermediate hinged triangular subassemblies through the same grammar adopted in Refs. [27–29, 38]. Accordingly, an allowed action consists of three steps: (i) selecting a node not yet connected by existing truss elements—such nodes are referred to as *inactive* to distinguish them from previously selected *active* nodes; (ii) selecting a truss element that is already part of the current layout; and (iii) applying the appropriate legal operator depending on the relative position of the selected node with respect to the chosen element. Two operators (grammar rules) are considered. As illustrated in Fig. 1, the $\mathcal{D}$ operator introduces the new node and connects it to the current structure without removing any existing elements, while the $\mathcal{T}$ operator removes the selected element before linking the new node. In both cases, new connections avoid intersections with existing elements.

## Computational framework

### Optimal truss design via standard Monte Carlo tree search

Monte Carlo tree search is a decision-time planning RL algorithm [31], leveraging two main principles: (i) approximating action-values through random sampling of simulated environment trajectories, and (ii) using these estimates to guide exploration of the search space, progressively steering the search toward highly rewarding trajectories. In the context of optimal truss design, MCTS incrementally grows a search tree, where nodes represent design configurations and edges encode layout modifications produced by admissible actions. Through repeated traversal and expansion of this tree, the algorithm learns a control policy, referred to as the *tree policy*, which is continuously refined using value estimates accumulated from previous training runs (or episodes).

**Fig. 2:**
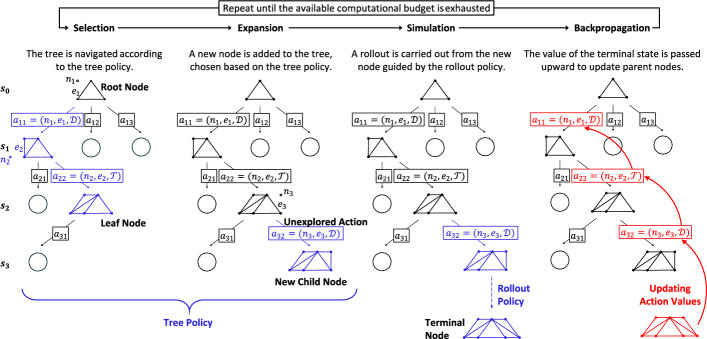
Schematic representation of the optimal truss design problem formalized as an MDP and solved through MCTS, with grammar rules guiding the process

As outlined in Fig. 2, each MCTS episode consists of four phases [31]. (i) *Selection*: starting from the root node (initial state), the algorithm descends the tree by selecting child nodes according to the tree policy, typically based on a UCT rule [39]. At each branching point, the child with the highest UCT score is selected, guiding the process toward a leaf node. (ii) *Expansion*: if the selected leaf is neither terminal nor fully expanded, one of its unexplored actions is used to create a new child node, thereby enlarging the tree and exploring new states. (iii) S*imulation*: a sequence of actions is executed from the newly added or selected leaf, until a terminal state ${s}_{T}$ is reached; these actions are sampled from a *rollout policy* that relies on randomized shuffling of generated children and on-demand feasibility checks. The resulting terminal reward provides a Monte Carlo trial that reflects the value of the simulated trajectory traversing the tree. (iv) *Backpropagation*: the reward obtained at the terminal node ${s}_{T}$ is propagated back through all visited nodes, updating their action-value ${Q}^{\pi }(s,a)$ for the corresponding state–action pairs.

Monte Carlo tree search offers several advantages owing to its incremental, real-time, sample-based value estimation. First, it excels in environments with delayed rewards, efficiently exploring large design spaces despite limited feedback. This makes it particularly suitable for progressive construction scenarios, where intermediate layouts often differ significantly from the final design. Second, MCTS builds a partial lookup table of action-value estimates only for state-action pairs encountered along promising trajectories, thereby eliminating the need to approximate a global value function. Accordingly, MCTS produces an asymmetric search tree that reflects valuable decision patterns and offers insights into the underlying design space.

### Design space exploration: upper confidence bounds for trees

The UCT formula is a widely used selection strategy within the MCTS framework. It effectively guides the exploration of large and complex decision spaces by balancing two competing criteria: exploiting actions that have previously yielded high rewards and exploring less-visited actions that may lead to improved outcomes. By managing this trade-off, UCT enables MCTS to focus computational resources on promising regions of the search space while maintaining sufficient exploration to avoid missing optimal solutions under a constrained computational budget.

In this work, we employ a modified *MixMax* UCT formulation [40], which computes the UCT score of the $j$ th child node of ${s}_{t}$ as follows:

6 $${\mathrm{UCT}}_{j}^{\alpha \beta }=\left(1-\alpha \right)\left[\frac{(1-\beta ){v}_{j}^{\Sigma }}{{n}_{j}}+\beta {v}_{j}^{\mathrm{best}}\right]+\alpha \sqrt{\frac{2\mathrm{log}\sum_{l}{n}_{l}}{{n}_{j}}},$$

where ${v}_{j}^{\Sigma }$ denotes the Monte Carlo estimate of the total accumulated reward obtained by traversing the tree through the $j$ th child node ($j=1,\dots ,J$, with $J$ being the total number of children). This quantity corresponds to the sum of all terminal rewards ${r}_{T}$ collected when the node is visited. The term ${v}_{j}^{\mathrm{best}}$ denotes the highest reward obtained by passing through the $j$ th child node. The quantity ${n}_{j}$ represents the number of episodes in which the path passes through the $j$ th child, whereas $\sum_{l}{n}_{l}$ denotes the total visit count of the parent node ${s}_{t}$, obtained by summing the visit counts of all its children. The hyperparameter $\alpha \in \left[\mathrm{0,1}\right]$ controls the exploitation–exploration balance, with the first and second terms representing the exploitation and exploration components, respectively. The hyperparameter $\beta \in \left[\mathrm{0,1}\right]$ regulates how exploitation is distributed between regions of the tree that consistently yield high average rewards and those that contain the single best observed reward. The quantities ${v}_{j}^{\Sigma }$, ${n}_{j}$, and, when applicable, ${v}_{j}^{\mathrm{best}}$ are updated at the end of every training episode.

By setting $\beta =0$ in Eq. (6), we retrieve the $\alpha$*-only* formulation used in [29]:

7 $${\mathrm{UCT}}_{j}^{\alpha }=\left(1-\alpha \right)\frac{{v}_{j}^{\Sigma }}{{n}_{j}}+\alpha \sqrt{\frac{2\mathrm{log}\sum_{l}{n}_{l}}{{n}_{j}}}.$$

It is worth noting that, for $\alpha =0.5$, the $\alpha$*-only* formulation coincides with the classical UCT expression with a unit exploration constant, which is the configuration that theoretically optimizes the exploitation–exploration trade-off in the multi-armed bandit problem when rewards are normalized between 0 and 1 [41, 42].

The rationale underlying the $\alpha$-only formulation is that branches of the search tree that yield better solutions on average are more likely to contain the optimal configuration. However, as noted by Garayalde et al. [29], this assumption may cause the algorithm to overlook branches that actually contain the global optimum. This limitation becomes particularly evident when the tree exhibits a high branching factor near the root, since the optimal solution may be hidden among many suboptimal alternatives. To address this issue, the MixMax selection strategy not only balances exploitation and exploration, but also explicitly regulates how exploitation itself is prioritized within the algorithm. This formulation, together with an appropriate choice of $\alpha$ and $\beta ,$ plays a crucial role in steering the search preventing premature convergence to local optima.

### Hierarchical-MCTS: a scalable search strategy

The MCTS framework for truss design [29] entails significantly lower computational costs compared to Q-learning, deep Q-learning, and Genetic Algorithms [27, 28]. However, like other truss design approaches, such as AlphaTruss [33], KR-based UCT [34], and Machine-Specified Ground Structures [26], its computational scalability is constrained by the curse of dimensionality. For MCTS, this limitation originates from the increasing number of admissible nodes in the design domain. Each additional node expands the set of valid truss topologies, leading to an exponential growth in the branching of the search tree and, accordingly, to substantial computational costs.

**Fig. 3:**
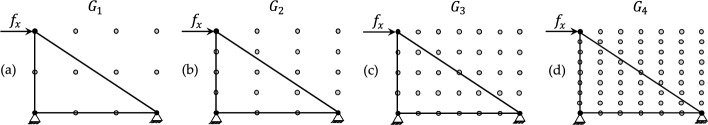
The input grids: **a** coarse grid ${G}_{1}$; **b** fine grid ${G}_{2}$; **c** finer grid ${G}_{3}$; **d** the finest grid ${G}_{4}$

To address this limitation, we introduce the H-MCTS method. Although the term *Hierarchical MCTS* also appears in [42], its purpose there differs fundamentally from our formulation. Here, the hierarchy refers to a sequence of grid refinements, with ${G}_{k}$ denoting the grid at stage $k=1,\dots ,K$. The optimization Problem (1) and the initial seed configuration are preserved across all stages, but they are embedded within progressively denser grid-world environments. In this study, we set $K=4$, empirically identified as a suitable compromise between solution quality and computational cost. Preliminary experiments have shown that fewer levels may not sufficiently refine the promising regions of the design space, whereas additional levels provide only marginal improvements while increasing computational time. Nevertheless, the number of refinement levels can be adjusted depending on the problem complexity and the available computational budget. However, the number of refinement levels can be adjusted according to problem complexity and computational budget. As illustrated in Fig. 3a–d, we consider four levels, from the coarse grid ${G}_{1}$ to the finest grid ${G}_{4}$.

**Fig. 4:**
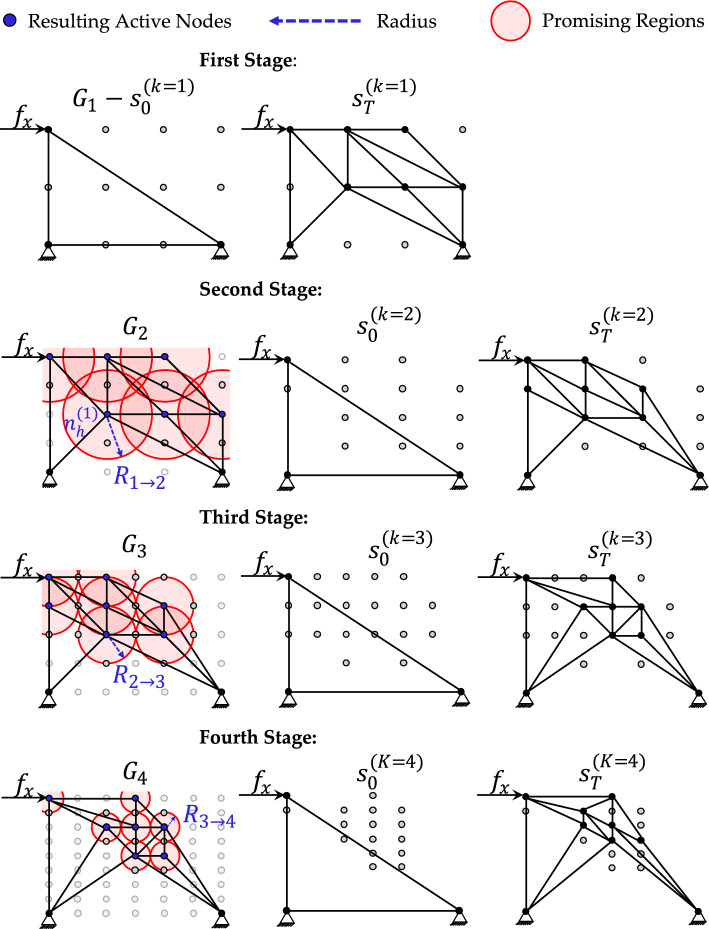
Hierarchical grid-refinement strategy. The process begins on the coarse grid ${G}_{1}$, where MCTS identifies active nodes to define circular refinement regions for the finer grid ${G}_{2}$. The procedure is repeated across grids ${G}_{3}$​ and ${G}_{4}$, progressively narrowing the design space

As shown in Fig. 4, the first-level MCTS begins on the uniform coarse grid ${G}_{1}$ and is executed for ${N}_{k=1}$​ episodes over the planning horizon $(0,T)$, using a specific tree policy ${\mathrm{UCT}}^{(k=1)}$ with parameters ${\alpha }_{k=1}$ and ${\beta }_{k=1}$. This produces an optimal policy ${\pi }_{k=1}$​, which traverses the search tree from the root state ${s}_{0}^{(k=1)}$​ to the terminal state ${s}_{T}^{(k=1)}$​, yielding a truss configuration that minimizes the design objective $f_{{k = 1}} {:} = \left\| {{\mathbf{U}}\left( {{{\Omega }}_{{k = 1}} } \right)} \right\|_{\infty }$ on ${G}_{1}$​. The $H$ active nodes present in ${s}_{T}^{(k=1)}$, denoted by ${n}_{h}^{(k=1)}$​ for $h=1,\dots ,H$, ​are collected in the set ${A}_{k=1}$. For each active node in ${A}_{k=1}$​ (excluding those serving as structural supports), a circular *refinement region* $\mathcal{C}$ is defined in ${G}_{2}$, centered at each ${n}_{h}^{(k=1)}$​ with radius ${R}_{1\to 2}$, determined from the node spacing​. Only inactive nodes located within or on the boundary of these regions are retained; all other inactive nodes are removed.

The second stage applies the same procedure to the grid ${G}_{2}$, which now contains only the regions identified as promising. A new MCTS run is performed with stage-specific parameters, generating a truss configuration ${s}_{T}^{(k=2)}$ along with its corresponding set of active nodes ${A}_{k=2}$. These nodes then serve to define smaller refinement zones in the next grid ${G}_{3}$​, further narrowing the design space. This process continues in subsequent stages, each time reducing the refinement radius. The final candidate solution is selected from all stages, based on the lowest achieved design objective.

### Structural adaptation under moving loads and damage scenarios

Rapid structural adaptation to accommodate evolving conditions, caused by moving loads and compromised truss elements, is enabled in H-MCTS through an offline–online strategy.

#### Preliminary offline phase

**Fig. 5:**
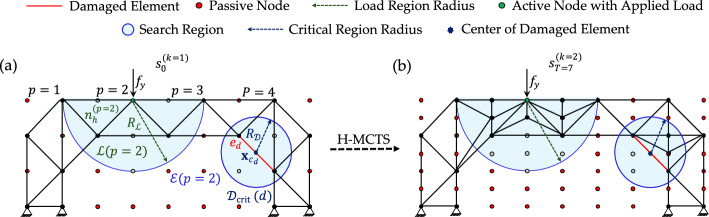
Exemplary offline data generation: **a** definition of the search region from load and critical regions; **b** optimized truss configuration obtained by H-MCTS at stage $k=2$ and terminal state $T=7$

In the case of a moving load scenario, we assume $P$ offline load positions, each identified by an active node ${n}_{h}^{(p)}$, where a static load ${f}_{y}$​ is applied sequentially for $p=1,\dots ,P$. As illustrated in Fig. 5 for an exemplary case with $P=4$, a circular *load region* $\mathcal{L}(p)$ is defined for each load position. The figure shows $\mathcal{L}(p=2)$, centered at ${n}_{h}^{(p=2)}$​ with a predefined radius ${R}_{\mathcal{L}}$​.

Similarly, if any element ${e}_{m}$, $m=1,\dots ,M$,​ with $M$ denoting the total number of elements, becomes damaged—meaning that any of its Young’s modulus ${E}_{m}$, yield strength ${\sigma }_{y,m}$, or cross-sectional area decreases—a circular *damaged region* $\mathcal{D}(d)$ is defined. This region is centered at the midpoint coordinate of the damaged element ${e}_{d}$​, with a chosen radius ${R}_{\mathcal{D}}$​. A damaged region is also termed *critical region*, denoted ${\mathcal{D}}_{\mathrm{crit}}(d)$, if the stress in element ${e}_{d}$ exceeds the yield strength ${\sigma }_{y,d}$​ or the buckling threshold ${\sigma }_{\mathrm{cr},d}$​ (in compression) for the current load configuration. The set of activated damaged elements is denoted with $D$.

In the presence of both moving loads and damage, the *search region* $\mathcal{E}(p)$ is defined as the union of load region $\mathcal{L}(p)$ and all critical regions ${\mathcal{D}}_{\mathrm{crit}}(d)$. Although this union may consist of multiple disconnected areas, it is treated as a single search region. As shown in Fig. 5a, all nodes lying outside $\mathcal{E}(p=2)$ are considered *passive* and are excluded from the optimization. Subsequently, as illustrated in Fig. 5b, H-MCTS is applied only to the inactive nodes located within or on the boundary of $\mathcal{E}(p)$, reinforcing both $\mathcal{L}(p)$ and ${\mathcal{D}}_{\mathrm{crit}}(d)$. This produces an optimized structural layout adapted to the current load position and any potential damage scenario. At the end of each stage $k$, a safety check is performed for every element ${e}_{m}$, $m=1,\dots ,M$,​ within ${s}_{T}^{(k)}$, verifying that stresses remain below the yield strength ${\sigma }_{y,m}$ or the buckling threshold ${\sigma }_{\mathrm{cr},m}$ (in compression).

The construction of the offline database requires performing an H-MCTS optimization for each considered load position and potential damage scenario. Although this precomputation stage may involve a non-negligible computational effort, this does not affect the online deployment phase. Moreover, optimizations associated with different load positions are mutually independent and can therefore be executed in parallel, enabling scalability to structures characterized by a higher number of loading or damage scenarios. Once the database is populated, the stored policies can be exploited online to rapidly synthesize near-optimal configurations with low computational cost.

Algorithm 1 outlines the offline pre-computation phase. Whenever an improved objective is found $\left({f}_{k}<{f}^{*}(p)\right)$ and all checks are satisfied, ${\pi }^{*}\left(p\right)$ is updated and stored in database $\mathcal{D}\mathcal{B}$ with ${f}^{*}\left(p\right)$.

#### Online adaptation phase

The online adaptation phase aims to synthesize a near-optimal structural layout for previously unseen load locations. This is achieved without executing a full H-MCTS optimization; instead, the method relies solely on offline H-MCTS runs. First, the two neighboring load positions are identified to determine the loading bracket, i.e., the pair of active nodes $\left({n}_{h}^{\left({p}^{-}\right)},{n}_{h}^{\left({p}^{+}\right)}\right)$ between which the actual load location lies, and for which precomputed policies are already available. The load is decomposed into equivalent nodal forces, denoted by ${f}_{y}^{(-)}$ and ${f}_{y}^{(+)}$, applied to the active nodes forming the loading bracket. The magnitudes of these forces are obtained by reducing the original load proportionally to the distance from the actual application point.

##### Algorithm 1: Offline pre-computation phase


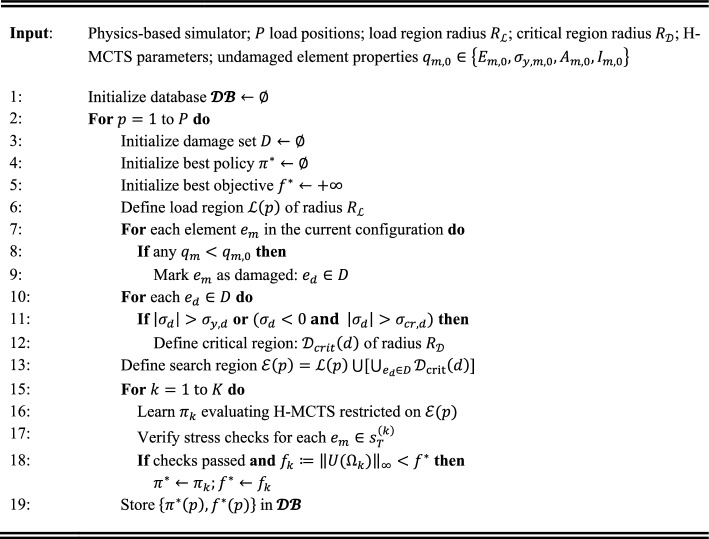


**Fig. 6:**
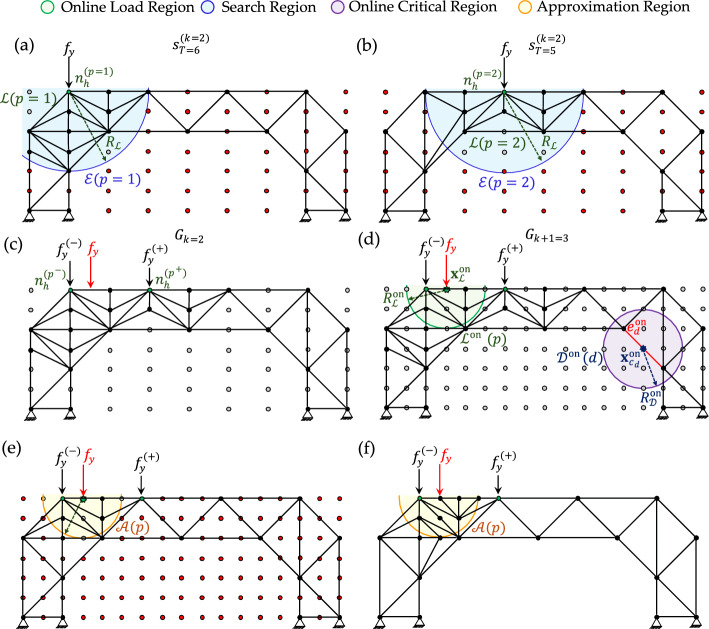
Exemplary online adaptation: **a**, **b** retrieval of stored configurations for load positions $p=1$ and $p=2$; **c** construction of the combined structure under rule-based merging; **d** mapping to grid ${G}_{k+1}$ and definition of the online load region​, with no activation of the critical region; **e** definition of the approximation region by removing passive nodes and external members; **f** generated near-optimal truss

Figure 6a, b illustrate an exemplary retrieval of stored offline results associated with load positions $p=1$ and $p=2$, respectively. A rule-based procedure is then used to construct the combined structure, as shown in Fig. 6c. In this step, the elements belonging to the initial configuration (see Fig. 5a) are preserved, while intersections between truss members from the two binary configurations are avoided. Whenever intersections occur, priority is given to the configuration closest to the applied load ${f}_{y}$. The retained members are selected so that the resulting configuration remains kinematically admissible, avoiding the loss of triangular substructures. Once obtaining the combined structure, its grid ${G}_{k}$ is mapped onto a higher-resolution grid ${G}_{k+1}$, and an *online load region* ${\mathcal{L}}^{\mathrm{on}}(p)$ is defined (see Fig. 6d). In the example shown, the stress ${\sigma }_{d}^{\mathrm{on}}$ does not exceed either threshold; therefore, no *online critical region* ${\mathcal{D}}_{\mathrm{crit}}^{\mathrm{on}}(d)$ is activated or included in the following steps.

Let us call a*pproximation region* the union of the online load region and all online critical regions:

8 $$\mathcal{A}\left(p\right)={\mathcal{L}}^{\mathrm{on}}\left(p\right)\cup \left[\bigcup_{{e}_{d}\in D}{\mathcal{D}}_{\mathrm{crit}}^{\mathrm{on}}\left(d\right)\right].$$

As shown in Fig. 6e, all inactive nodes located outside $\mathcal{A}(p)$ are marked as *passive* and removed from the optimization process. Only the nodes lying within or on the boundary of $\mathcal{A}(p)$ are retained. Similarly, all truss members outside $\mathcal{A}(p)$ are discarded, except for those belonging to the initial configuration (see Fig. 5a). Members that lie within $\mathcal{A}(p)$ or intersect its boundary are preserved. If necessary, additional connections are created between active nodes to form triangular patterns that ensure structural stability. Within $\mathcal{A}(p)$, generative grammar rules are applied subject to the maximum volume constraint ${V}^{\mathrm{max}}$ and, potentially, additional local restrictions, such as a maximum allowed element length ${L}^{\mathrm{max}}$. The resulting layout, as exemplified in Fig. 6f, represents a near-optimal truss configuration generated efficiently from previously stored computations for unseen load positions (and potentially damage scenarios) while satisfying all stress checks.

## Results

This section presents the computational results for a series of truss optimization problems. Section “Comparing alternative tree search selection strategies” compares the performance of alternative UCT-based selection strategies. Section “Efficiency and solution quality of H-MCTS” evaluates the proposed H-MCTS framework and compares it with its standard, non-hierarchical counterpart. Sections “Progressive construction and load-induced morphing” and 0 examine a bridge-like truss configuration for load-induced morphing and for structural adaptation in the presence of damage, respectively. Across the numerical experiments, performance is assessed in terms of design quality, learning dynamics, and computational cost. All experiments have been implemented in Python on a personal computer equipped with an Intel® Core™ i7-1165G7 CPU @ 2.80 GHz and 16 GB RAM.

### Comparing alternative tree search selection strategies

The UCT formulations introduced in Sect. “Design space exploration: upper confidence bounds for trees” are assessed on Case Study A, which has been previously examined in Refs. [26–28] and is summarized in Table 1. The domain size is expressed as $(y\times x)$, where $y$ denotes the number of rows and $x$ the number of columns in the nodal grid. The planning horizon is denoted by $T$, representing the maximum number of states explored by the algorithm. Two selection strategies are considered: the $\alpha$-only and MixMax formulations, given respectively by Eqs. (7) and (6). Each truss element is assigned dimensionless Young’s modulus $E={10}^{4}$, and a cross-sectional area $A=1$. The structure is subjected to a dimensionless concentrated load of magnitude $10$, while self-weight is neglected.

**Table 1:** Case study A—problem setup

Domain size $(y\times x)$	Planning horizon $(T)$	Max volume constraint ${(V}^{\mathrm{max}})$
$5\times 5$	$4$	$480$

**Fig. 7:**
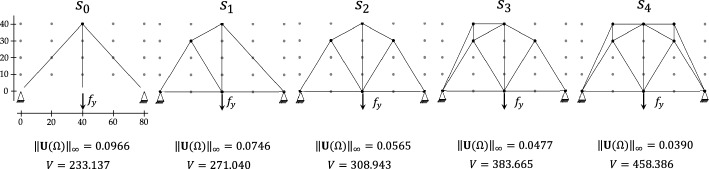
Case study A—learned sequence of structural configurations under MixMax selection strategy

Figure 7 illustrates the sequence of feasible designs generated from the learned optimal policy using the MixMax selection strategy with parameters $N=800$, $\alpha =0.1$, and $\beta =0.75$. For each configuration along the trajectory from the initial state ${s}_{0}$ to the terminal state ${s}_{T}$, we report the corresponding maximum absolute displacement ${\Vert \mathbf{U}(\Omega )\Vert }_{\infty }$ and structural volume $V$. For this benchmark case, the cardinality of the state space, defined as the number of valid terminal states obtained through an exhaustive search, is $\mathrm{1,433,800}$. The associated optimal objective ${\Vert \mathbf{U}(\overline{\Omega })\Vert }_{\infty }$​ is adopted as the ground truth for evaluating the MCTS performance.

Table 2 summarizes the MCTS results in terms of structural displacement trends, design performance, and computational cost for the two UCT formulations, based on five independent training runs. The displacement trend charts illustrate the evolution of the design objective ${\Vert \mathbf{U}(\Omega )\Vert }_{\infty }$​ as the number of training episodes increases. The mean displacement for the best-performing $\alpha$ value is shown as a solid line, while the shaded area denotes the one-standard-deviation interval. The value of $\beta$ for ${\mathrm{UCT}}_{j}^{\alpha \beta }$ is kept fixed based on a prior parameter investigation. The dashed line marks the global optimum from exhaustive search. The MixMax strategy converges to the global optimum within $400$ episodes, whereas the $\alpha$-only strategy does not reach it even after $\mathrm{10,000}$ episodes. The required number of episodes depends on the complexity of the case study and has been determined through an initial extended training run to asses convergence.

**Table 2:** Case study A—MCTS results for the MixMax and $\alpha$-only selection strategies, averaged over five training runs

UCT	Displacement trend	Design performance	Computational cost
MixMax policy			
$\alpha$-only policy			

The displacement trends depict the evolution of the design objective during training, shown as the average value (solid line) with its one-standard-deviation credibility interval (shaded area) and the corresponding global minimum (dashed line). Design performance is assessed monitoring the evolution of the objective ratio (%) relative to the global optimum. The computational cost is quantified in terms of the average number of FE evaluations for different values of the $\alpha$ parameter

Design performance is further assessed in terms of objective ratio, defined as the percentage ratio between the global optimum ${\Vert \mathbf{U}(\overline{\Omega })\Vert }_{\infty }$​ and the displacement ${\Vert \mathbf{U}(\Omega )\Vert }_{\infty }$ achieved by the learned policy. This metric quantifies the proximity of the maximum absolute displacement at each episode to the optimal objective. The curves corresponding to different values of $\alpha$ represent the averaged objective ratio over independent training runs, thereby providing insights into its impact.

The computational cost is quantified in terms of average number of FE evaluations. In this study, the strategy used to count FE evaluations differs from that adopted by Garayalde et al. [29]. In their work, FE analyses were performed only at the terminal node ${s}_{T}$, after its selection. In contrast, we perform FE evaluations for every newly populated node that passes the feasibility checks. Consequently, each intermediate state undergoes FE analyses, enabling us to monitor the progressive objective improvement from the initial configuration ${s}_{0}$​ to the terminal state ${s}_{T}$. In this way, MCTS receives richer information during backpropagation, strengthening the learning signals. Although this increases the total number of FE evaluations, the overall computational cost is alleviated by the proposed hierarchical approach, as demonstrated in the following section.

Table 3 reports the values of objective ratio; percentile score, which measures the ability of the algorithm to navigate the search tree (for example, a percentile score of $95\%$ indicates that the final design ${s}_{T}$​ performs better than $95\%$ of all configurations identified through exhaustive search); and average elapsed time, which provides additional insight into computational effort.

**Table 3:** Case study A—objective ratio, percentile score, and average elapsed time for the two tree policies

UCT	$\alpha$	$\beta$	${\Vert \mathbf{U}(\overline{\Omega })\Vert }_{\infty }$	Objective ratio $[\%]$	Percentile score $[\%]$	Elapsed time $[s]$
MixMax policy	$0.1$	$0.75$	$0.039$	$100$	$100$	$23$
$\alpha$-only policy	$0.5$	—		$88.783$	$99.962$	$320$

The MixMax policy outperforms the $\alpha$-only formulation in terms of both design performance and search efficiency, while also requiring substantially lower computational effort. MCTS with ${\mathrm{UCT}}_{j}^{\alpha \beta }$ achieves an objective ratio of $100\%$, i.e., the global optimum, whereas ${\mathrm{UCT}}_{j}^{\alpha }$ attains a lower performance. Nevertheless, ${\mathrm{UCT}}_{j}^{\alpha \beta }$ requires an average of $780$ FE evaluations and about $23$ seconds, while ${\mathrm{UCT}}_{j}^{\alpha }$ requires $\mathrm{19,570}$ FE evaluations and about $320$ seconds. Compared with Garayalde et al. [29], the difficulty in their study in recovering the optimal layout, resulting instead in a close suboptimal solution, can therefore be attributed to the exclusive use of ${\mathrm{UCT}}_{j}^{\alpha }$​.

The superior performance of the MixMax policy can be attributed to its ability to​ enhance tree navigation by balancing the trade-off between the average reward and the best-seen reward. This capability becomes especially relevant in problems with large branching factors, where the global optimum may lie within regions with low average reward and thus be overlooked by an $\alpha$-only policy​. It is also worth highlighting that ${\mathrm{UCT}}_{j}^{\alpha \beta }$​ tends to achieve its best performance for values close to $\beta =1$, as the exploitation term is fully concentrated on the best-seen reward.

### Efficiency and solution quality of H-MCTS

In this section, we focus on several case studies, all addressed using H-MCTS with the MixMax tree policy. The parameters employed at each H-MCTS stage are reported in Table 4. For all case studies, we adopt the same material properties and load magnitude used in Sect. “Comparing alternative tree search selection strategies”. The effectiveness of H-MCTS is then assessed through comparison with the baseline MCTS.

**Table 4:** Case studies (B–F)—H-MCTS parameters at each stage; parameter $\beta$ is set to 1 for all stages

Case study	Stage $k$	Grid ${G}_{k}$	${\alpha }_{k}$	Episodes ${N}_{k}$
B	$1$–$4$	$3\times 9$; $5\times 17$; $11\times 21$; $26\times 21$	$0.2$; $0.1$; $0.05$; $0.05$	$1000$ each
C	$1$–$4$	$4\times 3$; $7\times 5$; $11\times 11$; $16\times 21$	$0.3$; $0.1$; $0.05$; $0.05$	$150$; $400$; $600$; $800$
D	$1$–$3$	$5\times 3$; $9\times 5$; $17\times 9$	$0.3$; $0.1$; $0.05$	$400$; $1000$; $1400$
E	$1$–$3$	$5\times 5$; $9\times 9$; $17\times 9$	$0.5$; $0.1$; $0.05$	$1000$; $1500$; $2000$
F	$1$–$3$	$7\times 7$; $13\times 13$; $13\times 25$	$0.1$; $0.1$; $0.05$	$1000$ each

**Fig. 8:**
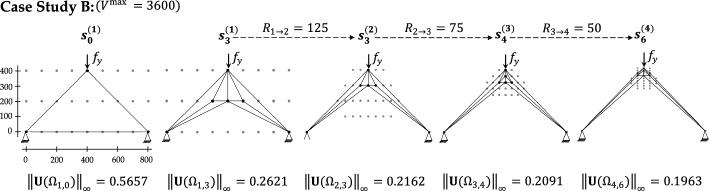
Case study B—progressive refinement of truss configurations across four H-MCTS stages, from the seed layout to the stage-wise optimal designs, under a fixed maximum allowed volume

Figure 8 illustrates how the seed configuration and the corresponding objective improve from stage 1 to stage 4 for Case Study B. At each stage, once the terminal state ${s}_{T}^{(k)}$ is reached for $k=1,\dots ,4$, the resulting structural layout exhibits symmetric geometry, reflecting the symmetric boundary conditions and thereby indicating the quality of the synthesis process.

**Fig. 9:**
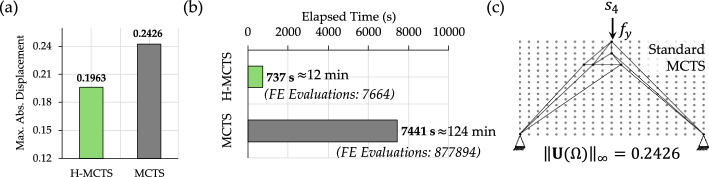
Case study B—comparative performance of H-MCTS and standard MCTS: **a** maximum absolute displacement objective; **b** elapsed time and number of required FE evaluations; **c** final truss configuration obtained by standard MCTS on the $17\times 21$ grid, illustrating an asymmetric solution

Overall, the quality of the structural configuration tends to improve as the number of nodes available in the design domain increases. For example, applying single-stage MCTS to Case Study B on a finer $17\times 21$ grid (see Fig. 9c) reduces the maximum displacement from $0.2621$ on the coarse $3\times 9$ grid (see Fig. 8) to $0.2426$. However, Fig. 9c also shows that the structural layout generated by standard MCTS does not preserve symmetry. This asymmetry results from the suboptimal exploration in high-resolution design spaces. Moreover, attempts to apply standard MCTS on finer grids exhibit premature termination or failure to complete the optimization process [29]. The proposed H-MCTS framework overcomes these limitations through a stable and progressive exploration of increasingly finer grids, ultimately reaching a resolution of $26\times 21$. As shown in Fig. 9a, H-MCTS achieves a $19.1\%$ reduction in the maximum absolute displacement compared to single-stage MCTS. In addition, Fig. 9b demonstrates that the computational time required by H-MCTS is substantially lower, yielding a reduction of nearly two hours despite the finer grid.

**Fig. 10:**
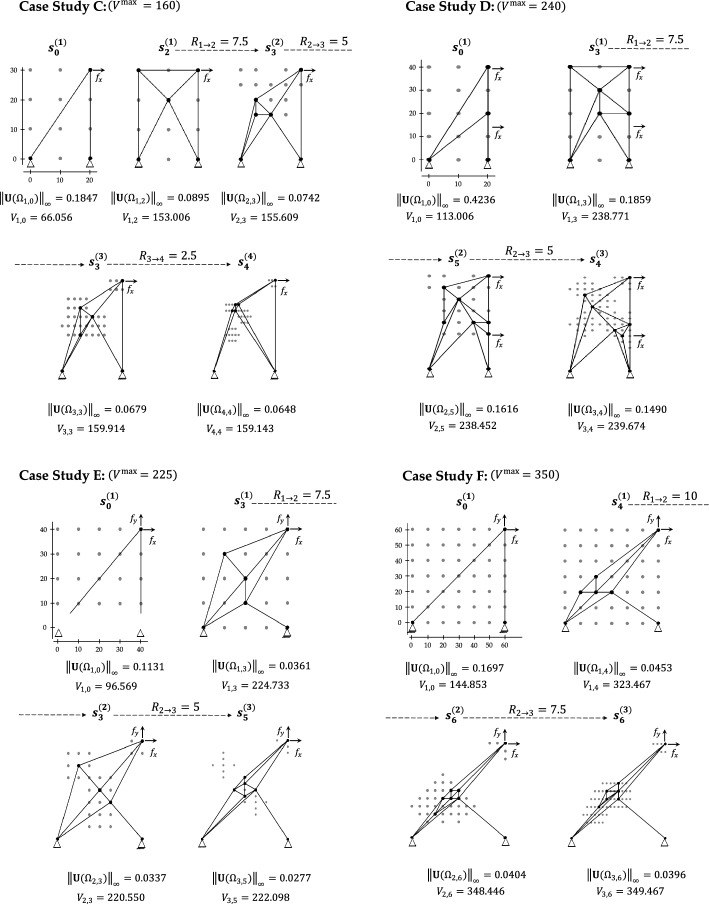
Case studies (C–F)—initial layouts and synthesized solutions at the end of each H-MCTS stage

The generalizability of H-MCTS is further assessed on benchmark Case Studies (C–F), adapted from previous research on truss optimization using MCTS, Q-learning, and deep Q-learning [27, 28, 33]. The problem specifications for all case studies are shown in Fig. 10, in terms of discretized design grid, initial truss configuration, loading conditions, and maximum allowable volume. The hierarchical optimization results for these benchmarks are also reported in this figure, which displays the final truss configurations obtained at the end of each H-MCTS refinement stage together with the corresponding transition radii ${R}_{k\to k+1}$.

**Fig. 11:**
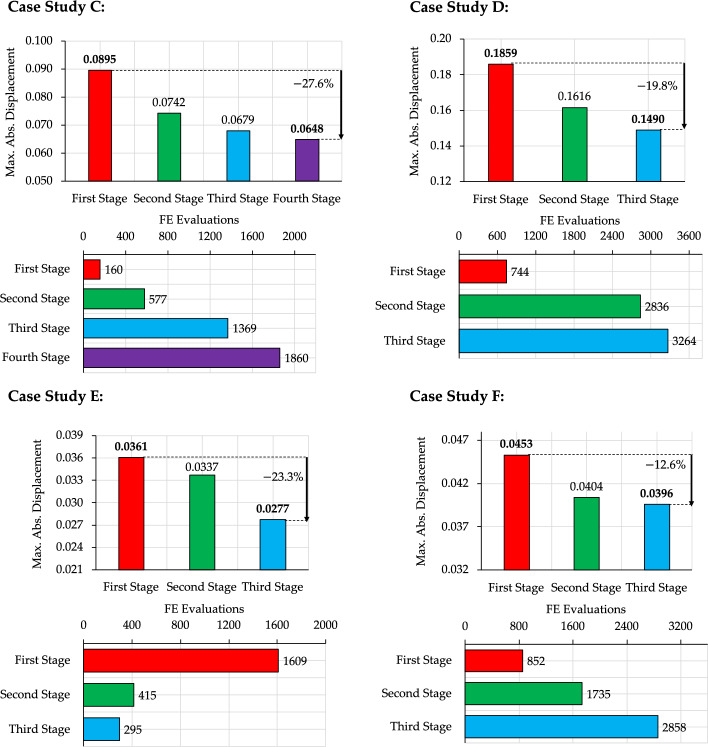
Case studies (C–F)—stage-wise reduction in the maximum absolute displacement objective and corresponding number of FE evaluations throughout the H-MCTS optimization process

Figure 11 presents the evolution of the design objective across successive H-MCTS stages for Case Studies (C–F). The results demonstrate a consistent improvement in the design objective as the search progresses through increasingly refined grids. Compared to standard MCTS, Q-learning, and deep Q-learning [27, 28, 33], the H-MCTS framework proves more effective, matching the performance of these methods in the first stage and surpassing them in the subsequent ones. The computational cost at each H-MCTS stage, in terms of number of FE evaluations, is also reported.

Case Study C exhibits the largest improvement, with the best policy ${\pi }^{*}$ at the fourth H-MCTS stage yielding a $27.6\%$ reduction in the design objective (see Fig. 11). For the other case studies, the best-performing configuration is generally achieved by the third stage. Moreover, for Case Study F, H-MCTS can not reproduce the first-stage optimal layout identified through exhaustive search [29], attaining an objective ratio of $92.72\%$. However, this still surpasses the $90.44\%$ achieved in [29], an early advantage attributed to the use of the MixMax tree policy. This initial shortfall is then addressed through subsequent refinements, ultimately resulting in a $12.6\%$ reduction in the maximum absolute displacement by the final stage.

As shown in Fig. 11, the computational effort generally increases with each optimization stage. This is expected, since refinements add inactive nodes within structurally promising zones, thereby increasing the branching factor of the search tree. Consequently, more FE evaluations are required to adequately explore the enlarged design space. For instance, in Case Study D, the number of inactive nodes increases from $11$ in the first stage to $22$ in the second and $60$ in the third. Similarly, the number of FE evaluations increases from $744$ in the first stage to $2836$ in the second and $3264$ in the third. These increases also reflect the presence of multiple competitive design alternatives, which makes it more challenging to discriminate between closely performing configurations.

Here, H-MCTS addresses this increasing complexity by progressively enhancing exploitation through reduced values of the $\alpha$ parameter at higher levels of the hierarchy (see Table 4), thereby narrowing the search toward the most promising branches of the tree. At the same time, $\beta$ is kept fixed at $1$ to reinforce the preference for actions associated with the best-observed rewards. These simple adjustments ensure that computational effort is progressively concentrated toward search paths that yield substantial reductions in the design objective.

**Fig. 12:**
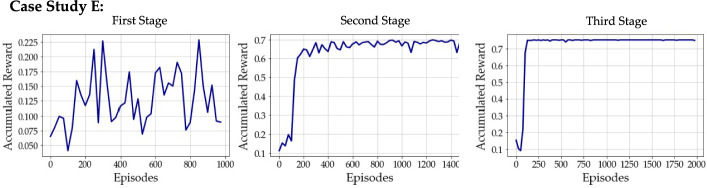
Case study E—evolution of the reward accumulated over training for the three consecutive stages. Results are averaged over batches of $25$ episodes

Interestingly, Case Study E exhibits a declining trend in the number of FE evaluations across successive stages (see Fig. 11)—namely, $1609$, $415$, and $295$ for the first, second, and third stages, respectively. This behavior is attributed to the low complexity of the promising regions identified through grid refinements. Indeed, the number of inactive nodes remains relatively low throughout the three stages ($22$, $26$, and $22$ in the first, second, and third stages, respectively), preventing an excessive growth in the number of design alternatives. This enables more focused exploration and accelerates convergence toward the optimal policy. As shown in Fig. 12, the learning process stabilizes after the first stage. Initially, the training features significant oscillations in the accumulated reward; however, the second stage displays a rapid and steady performance increase, indicating that the algorithm leverages the refined knowledge gained earlier more efficiently. The third stage further consolidates this improvement showing minimal variability.

Table 5 reports an exemplary phase-by-phase timing breakdown across the three hierarchical levels of Case Study D. The predominance of simulation time arises from the repeated validation of candidate actions. During rollouts, newly generated children are first subjected to geometric and constraint checks (such as action admissibility, domain conformity, and element-length requirements) [29]. If a child node satisfies these checks, it is then populated through FE assembly and solution to compute displacements and stresses. Because many candidates are evaluated and both feasibility checks and FE analyses are performed at each step of the rollout, the simulation phase emerges as the most time-consuming component of the optimization process.

**Table 5:** Case study D—timing breakdown for each H-MCTS phase across three hierarchical stages; the predominance of simulation time is highlighted in bold

MCTS phase	Elapsed time $[s]$		
	First stage	Second stage	Third stage
Selection	0.025	0.222	0.866
Expansion	0.517	1.549	8.229
**Simulation**	**11.223**	**57.857**	**196.414**
Backpropagation	0.009	0.235	0.598
Total	11.774	59.862	206.106

### Progressive construction and load-induced morphing

Another potential of MCTS-based strategies for truss design lies in their progressive nature [29], mimicking additive manufacturing processes [43]. This capability is demonstrated here through a bridge-like truss example. We consider truss members made of Eurocode IPE 80 profile (cross-sectional area $A=$ $7.64$ ${\mathrm{cm}}^{2}$, second moment of area $I=$ $8.49$ ${\mathrm{cm}}^{4}$, and radius of gyration ${r}_{g}=$ 1.05 $\mathrm{cm}$) in structural steel $\mathrm{S}235$, with mechanical properties: density $\rho =7850$ $\frac{\mathrm{kg}}{{\mathrm{m}}^{3}}$; yield strength ${\sigma }_{y}=235$ $\mathrm{MPa}$; and Young’s modulus $E=210$ $\mathrm{GPa}$.

**Fig. 13:**
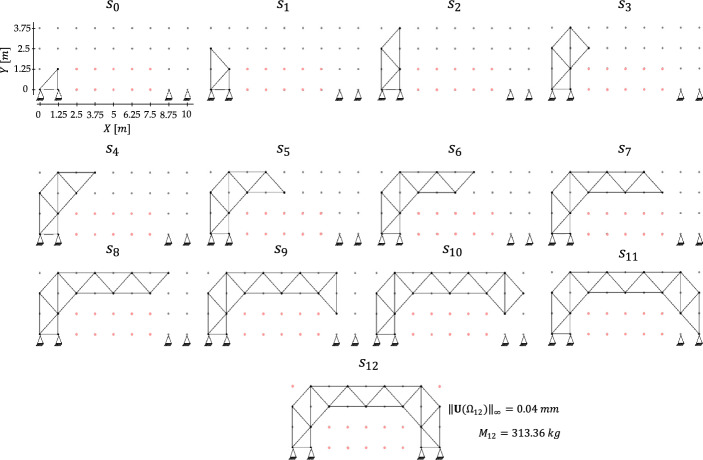
Bridge-like case study—sequence of truss configurations under self-weight, passive nodes in red

In contrast to the previous case studies, where the seed configuration covered the design domain in compliance with the assigned boundary conditions, the present setting allows the seed configuration to grow progressively. As shown in Fig. 13, the seed layout is not pre-connected to the target support node; rather, the structure must develop toward it through sequential assembly. This setup introduces additional complexity: the loading condition is not fixed, and the agent must evaluate the performance of intermediate construction stages by balancing the stiffness gained from newly added members against the corresponding increase in weight. Despite these changes, the objective remains to minimize the maximum absolute displacement for the final configuration.

Practical constraints reflecting fabrication, transportation, and on-site assembly limitations are incorporated by restricting the maximum volume of the structure and the maximum length of individual elements, as reported in Table 6. Moreover, a central passive area is introduced in the domain to represent the void below the deck.

**Table 6:** Bridge-like case study—design constraints, applied moving load, and extra mass allowance

Max. volume $({V}^{\mathrm{max}})$ $\left[{\mathrm{m}}^{3}\right]$	Max. element length ${(L}^{\mathrm{max}})$ $\left[m\right]$	Applied load ${(f}_{y})$ $\left[\mathrm{kN}\right]$	Extra mass $\left[\mathrm{kg}\right]$
0.046	2.5	49.05	47.1

Following the setup of Garayalde et al. [29], the algorithm is implemented using the ${\mathrm{UCT}}_{j}^{\alpha }$ selection scheme with $\alpha =0.3$, and the training is carried out over $1000$ episodes. The sequence of intermediate configurations from the initial state to the final design is shown in Fig. 13. The resulting terminal state ${s}_{12}$ corresponds to the global optimum obtained via exhaustive search. This configuration serves as the starting point for the subsequent morphing process.

Load-induced morphing is performed with reference to a point mass of $5000 \mathrm{kg}$, mimicking a moving vehicle. The objective is to improve the objective ratio by locally modifying the topology in the region close to the traveling load. To ensure these adjustments remain confined to the already developed structure, the central passive area is preserved, and the top-left and top-right corners of the domain are also enforced as passive, as illustrated for the terminal state ${s}_{12}$ in Fig. 13.

**Fig. 14:**
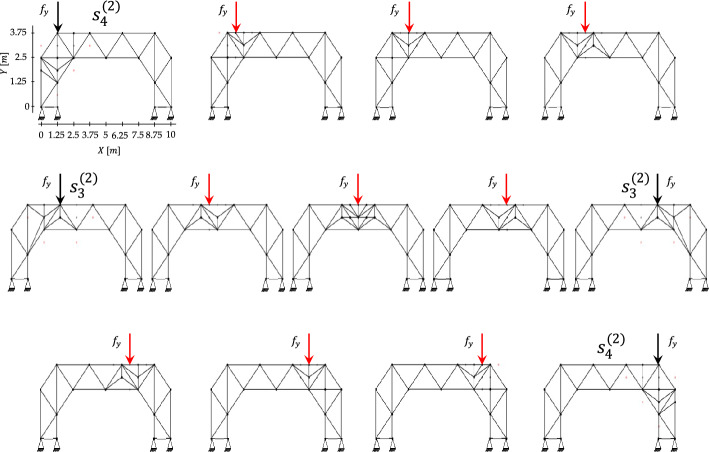
Load-induced morphing—results for each loading position, showing morphing of the truss topology under offline H-MCTS solutions (black arrows) and interpolated layouts (red arrows)

Morphing is achieved through the offline–online decoupling. Offline, the algorithm exploits the remaining mass available after progressive construction (see Table 6). Since the final configuration ${s}_{12}$ has a mass of $313.36 \mathrm{kg}$ and the maximum allowable mass is ${M}^{\mathrm{max}}=361.1 \mathrm{kg}$, a residual budget of $47.74 \mathrm{kg}$ is available for local reinforcement. These resources are exploited to respond to the point mass statically applied at four hinge positions along the deck (see Fig. 14), indexed as $p=1,\dots ,4$. For each loading case, H-MCTS is executed up to the second refinement level using a fine $7\times 9$ grid, with $\alpha =0.1$, $\beta =1$, and $1000$ episodes per level. The resulting optimal truss configurations form a database of binary solutions associated with the prescribed load locations.

In the online phase, an additional extra mass allowance of $47.1 \mathrm{kg}$ is introduced to enable further potential modifications, as clarified below. New layouts for previously unseen, intermediate load positions are generated by interpolating the two nearest offline solutions and subsequently projecting the result onto a refined $7\times 17$ grid.

The set of optimized and interpolated configurations is shown in Fig. 14, illustrating the morphing of the truss topology as the load travels along the deck. The layouts adapt by locally reinforcing the regions near the applied load while preserving the global structural form. Symmetric loading conditions lead to symmetric layouts, as well as to the same depth of the explored search tree in the offline phase (i.e., the number of decision steps required to reach the final designs).

Table 7 reports comparison results obtained through full H-MCTS optimizations performed on the same grid using $\alpha =0.05$, $\beta =1$, and $N=1000$. For each intermediate load location, we report the structural mass, the maximum absolute displacement, the elapsed time for the H-MCTS run, and the design accuracy of the interpolated layout relative to the corresponding H-MCTS solution. While each H-MCTS optimization requires approximately one to three minutes of computation, the interpolated designs are generated instantaneously, as they rely solely on combining and projecting the stored offline solutions. Across all cases, interpolation achieves an average accuracy of $88.83\%$, with the highest value of $90.87\%$ obtained at the symmetric load coordinates $(\text{2.5, 3.75}\text{ m})$ and $(\text{7.5, 3.75}\text{ m})$. The interpolated configuration corresponding to the central hinge (5, 3.75 m) is the only one that requires additional structural mass beyond the initial maximum allowable mass of ${M}^{\mathrm{max}}=361.1 \mathrm{kg}$. This is due to the need for supplementary members to redistribute internal forces and reduce the resulting displacement. It is important to note that without exceeding ${M}^{\mathrm{max}}$, the additional elements would simply increase the structural weight without reducing the maximum absolute displacement. For all the other cases, the interpolated configurations remain below the initial mass threshold ${M}^{\mathrm{max}}$.

**Table 7:** Load-induced morphing—performance comparison between interpolated and H-MCTS-derived truss configurations across all intermediate load positions

Intermediate load position	Mass $[kg]$ (${M}^{\mathrm{max}}$ = 361.1 kg)		Max. Abs. displacement $[\mathrm{mm}]$		Elapsed time $[\mathrm{min}]$	Design accuracy $[\%]$
	Approx	H-MCTS	Approx	H-MCTS	H-MCTS	
(1.875, 3.75)	357.91	360.16	0.92	0.81	1.37	88.62
(2.5, 3.75)	352.61	360.16	1.37	1.24	2.52	90.87
(3.225, 3.75)	358.13	357.57	1.83	1.64	3	90.01
(4.375, 3.75)	354.38	361.05	1.92	1.66	1.42	86.41
(5, 3.75)	404.24	357.44	1.71	1.50	1.58	87.64
(5.625, 3.75)	354.38	361.05	1.92	1.66	1.22	86.41
(6.875, 3.75)	358.13	357.57	1.83	1.64	3.43	90.01
(7.5, 3.75)	352.61	360.16	1.37	1.24	2.08	90.87
(8.125, 3.75)	357.91	360.16	0.92	0.81	1.32	88.62

### Damage-aware structural adaptation

With reference to the same bridge-like truss considered in the previous section, we now introduce the possibility of further structural adaptation under a representative damage scenario. In this study, the damage mechanism is assumed to reflect thermal degradation due to high-temperature exposure. Even in the absence of visible failure, elevated temperatures can compromise the internal microstructure of steel, resulting in reduced stiffness and strength. To capture this effect, the Young’s modulus and yield strength of the damaged members are reduced to $E=84 \mathrm{GPa}$ and ${\sigma }_{y}=141 \mathrm{MPa}$, respectively. We acknowledge that this representation of temperature-induced damage is simplified, as real effects may also involve additional phenomena such as residual stresses, creep, and geometric distortions.

To detect and address this degradation, the algorithm first identifies the damaged element and evaluates whether it is critical. If either the reduced yield strength is exceeded or, for members in compression, the critical buckling stress is exceeded, the damaged element is classified as critical. In the offline phase, if the damaged element is not critical, optimization proceeds as before, with a mass limit of ${M}^{\mathrm{max}}=361.1 \mathrm{kg}$. Conversely, if the element is classified as critical, this limit is relaxed by introducing the additional mass allowance of $47.1 \mathrm{kg}$ for localized reinforcement. The selection parameters, grid resolutions, and number of training episodes remain unchanged from the previous section. During the online approximation phase, in which two neighboring layouts are combined and further strengthening may be required in critical zones, the available mass budget is further increased to ${M}^{\mathrm{max}}=471 \mathrm{kg}$ to accommodate on-demand reshaping.

**Fig. 15:**
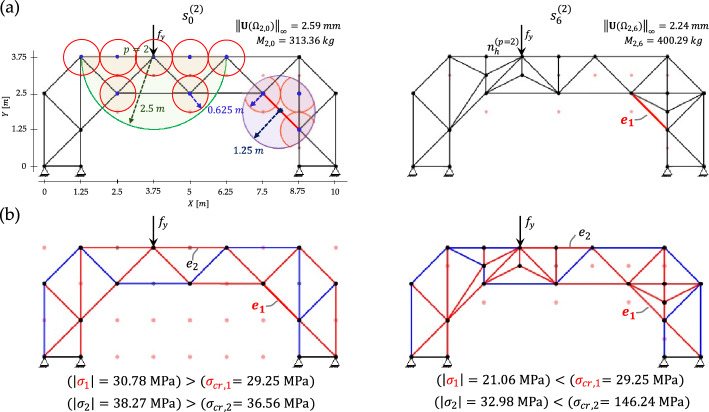
Damage-aware adaptation: **a** seed layout and second-stage optimized configuration under loading at node ${n}_{h}^{(p=2)}$, with details of the load and critical regions; **b** corresponding axial stress distributions, showing stress reduction achieved via reinforcement of damaged and slender elements

As shown in Fig. 15a for a pilot case, the damaged member ${e}_{1}$ exceeds its critical buckling stress for the load applied at node ${n}_{h}^{(p=2)}$. A circular critical region is defined around it, and offline H-MCTS is executed within both the load region and the critical region. The optimized configuration obtained at the second refinement stage achieves a $9.44 \%$ reduction in maximum absolute displacement relative to the coarse-grid outcome and a $13.70 \%$ reduction compared with the seed layout, demonstrating the effectiveness of localized refinement. Figure 15b presents the axial stress distributions for the initial and optimized configurations under the same loading condition, with tension and compression indicated by blue and red members, respectively. H-MCTS reinforces not only the damaged members but also intact slender elements that would otherwise buckle. In particular, the slender member ${e}_{2}$ violates its buckling limit in the seed configuration; H-MCTS subdivides it into shorter segments, increasing its capacity from $36.56$ to $146.24\text{ MPa}$.

**Fig. 16:**
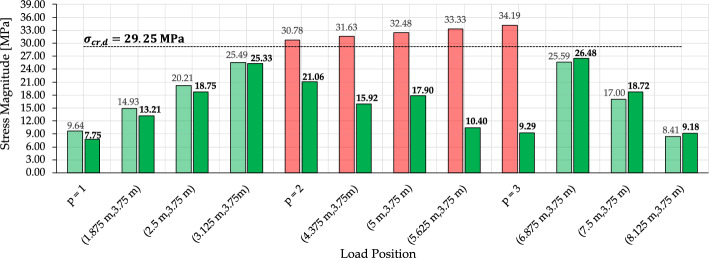
Damage-aware adaptation—stress magnitude in the damaged element before (light-colored bars) and after (dark-colored bars) adaptation for each load position

To evaluate the evolution of stresses in the damaged member as the load travels across the deck, Fig. 16 tracks the corresponding axial stress before and after optimization for each load position. The light-colored bars represent the stress magnitude in the damaged element for the initial configuration, whereas the dark-colored bars correspond to the optimized layouts obtained by either offline H-MCTS or online approximation, depending on the load case. The damaged element is classified as critical for loads applied from $p=2$ to $p=3$, which requires activation of the critical region in both the offline and online optimization phases. Accordingly, distinct offline solutions must be stored for scenarios without damaged elements and for those with an activated critical region. After optimization, the stress in the damaged element is substantially reduced for all critical cases. The stress is not reported for $p=4$ since the damaged element is under tension.

**Fig. 17:**
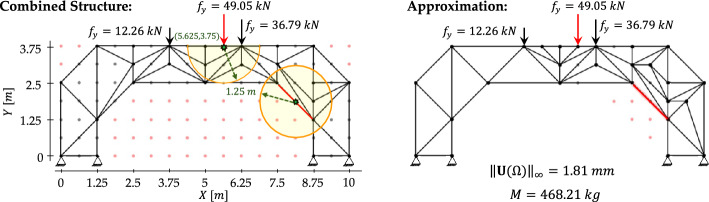
Damage-aware adaptation—approximated layout for an exemplary intermediate load case, with details of critical region reinforcement and the resulting performance improvement

An exemplary approximated layout for the intermediate loading position $(\text{5.625, 3.75}\hspace{0.17em}\mathrm{m})$ is shown in Fig. 17. This configuration is obtained by combining the two binary solutions optimized for loads applied at nodes ${n}_{h}^{(p=2)}$ and ${n}_{h}^{(p=3)}$. Since the damaged element ${e}_{1}$ is critical for this loading location, the online approximation region includes both the load and damaged regions. The available mass budget thus enables grammar-compliant structural modifications within these regions. The resulting design reduces the axial stress in the damaged element by $68.80 \%$ and decreases the maximum absolute displacement from $2.19$ to $1.82 \mathrm{mm}$.

**Fig. 18:**
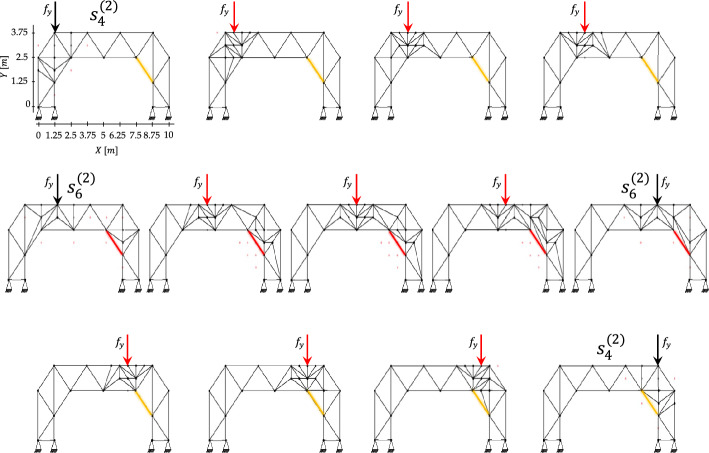
Damage-aware adaptation—optimized layouts corresponding to each load position. The damaged element is marked in red whenever the critical region is activated

The complete set of optimized configurations for all loading positions is shown in Fig. 18. Yellow-highlighted members denote damaged but non-critical elements, while red-highlighted members identify critical elements. When the critical region is not triggered, both H-MCTS and the interpolation procedure yield symmetric layouts; however, symmetry is no longer observed once the critical region is triggered. The largest displacement occurs for load position $p=2$, where it decreases from $2.59 \mathrm{mm}$ in the seed configuration to $2.24 \mathrm{mm}$ after optimization. The best-performing approximation corresponds to the load at $(\text{5.625, 3.75}\hspace{0.17em}\mathrm{m})$, shown in Fig. 17.

## Conclusions

This paper has introduced a Hierarchical Monte Carlo Tree Search (H-MCTS) framework for truss topology optimization that integrates grammar-guided actions, staged grid refinements, and an offline–online morphing strategy. The proposed approach enables efficient navigation of large design spaces while ensuring structural feasibility at every intermediate configuration.

The framework has been validated on benchmark problems drawn from previous research studies using classical MCTS [29], Q-learning [27], and deep Q-learning [28]. Across all examined case studies, the MixMax tree policy has consistently outperformed the $\alpha$-only formulation, demonstrating that emphasizing exploitation with $\alpha$ values around $0.1$ and $\beta$ equal to $1$ allows the discovery of high-quality designs with substantially fewer finite element evaluations. Moreover, the proposed hierarchical refinement strategy efficiently mitigates the curse of dimensionality that limits standard MCTS on dense grids and, in the refined stages, consistently surpasses its performance while significantly reducing the computational burden relative to baseline MCTS.

The framework has been further assessed on a bridge-like truss undergoing progressive construction, moving loads, and localized damage. The proposed offline–online morphing strategy has enabled rapid adaptation to shifting load positions based on offline precomputations. The resulting online-interpolated layouts have achieved an average accuracy of 88.8% relative to full H-MCTS solutions. Additionally, in the presence of material degradation, the damage-aware extension has successfully identified and reinforced critical regions, reducing stresses in compromised members by up to 72.8%.

Future developments may extend the framework to variable member profiles, potentially exploiting reusable component inventories to support circular and resource-efficient design strategies [44]. In a similar spirit, the approach could be generalized to three-dimensional domains, where deep learning–assisted policy networks may guide the search in significantly larger action spaces. Additional research directions include the integration of continuous-domain representations, for instance via kernel regression [34], as well as the incorporation of dynamic loading scenarios to enable adaptive structures capable of maintaining high performance under realistic, time-varying environments. A further promising direction involves extending the framework to account for uncertainties in material properties and external loads, allowing the synthesis of truss configurations that remain robust under varying operational conditions.

## Data Availability

No datasets were generated or analysed during the current study.

## References

[1] Campbell MI, Shea K. Computational design synthesis. AI EDAM. 2014;28(3):207–8. 10.1017/S0890060414000171.

[2] Rozvany GIN. A critical review of established methods of structural topology optimization. Struct Multidiscip Optim. 2009;37(3):217–37. 10.1007/s00158-007-0217-0.

[3] Bendsøe MP, Kikuchi N. Generating optimal topologies in structural design using a homogenization method. Comput Methods Appl Mech Eng. 1988;71(2):197–224. 10.1016/0045-7825(88)90086-2.

[4] Bendsøe MP. Optimal shape design as a material distribution problem. Struct Optim. 1989;1(4):193–202. 10.1007/BF01650949.

[5] Rozvany GIN, Zhou M, Sigmund O. Optimization of Topology in: advances in design optimization. Chapman & Hall; 1994.

[6] Allaire G, Bonnetier E, Francfort G, Jouve F. Numerische Mathematik Shape optimization by the homogenization method; 1997.

[7] Zhou M, Rozvany GIN. The COC algorithm, Part II: topological, geometrical and generalized shape optimization; 1991.

[8] Lewiński T, Zhou M, Rozvany GIN. Extended exact least-weight truss layouts-Part II: unsymmetric cantilevers. Int J Mech Sci. 1994;36(5):399–419. 10.1016/0020-7403(94)90044-2.

[9] Svanberg K. The method of moving asymptotes—a new method for structural optimization. Int J Numer Methods Eng. 1987;24:359–73.

[10] Osher S, Sethian JA. Fronts propagating with curvature-dependent speed: algorithms based on Hamilton-Jacobi formulations. J Comput Phys. 1988;79(1):12–49. 10.1016/0021-9991(88)90002-2.

[11] Sethian JA, Wiegmann A. Structural boundary design via level set and immersed interface methods. J Comput Phys. 2000;163(2):489–528. 10.1006/jcph.2000.6581.

[12] Osher S, Fedkiw R. Level set methods and dynamic implicit surfaces, vol. 153. 2003. 10.1007/B98879.

[13] Duysinx P, Van Miegroet L, Jacobs T, Fleury C. Generalized shape optimization using X-FEM and level set methods. Solid Mech Appl. 2006;137:23–32. 10.1007/1-4020-4752-5_3.

[14] Cho S, Ha SH, Kim MG. Level set based shape optimization of geometrically nonlinear structures. Solid Mech Appl. 2006;137:217–26. 10.1007/1-4020-4752-5_22.

[15] Allaire G, Jouve F. Coupling the level set method and the topological gradient in structural optimization. Solid Mech Appl. 2006;137:3–12. 10.1007/1-4020-4752-5_1.

[16] Rozvany GIN, Zhou M, Birker T. Generalized shape optimization without homogenization. Struct Multidiscip Optim. 1992;4(3):250–2. 10.1007/BF01742754.

[17] Deb K, Gulati S. Design of truss-structures for minimum weight using genetic algorithms. Finite Elem Anal Des. 2001;37(5):447–65. 10.1016/S0168-874X(00)00057-3.

[18] Saka MP, Hasançebi O, Eser H, Geem ZW. Historical evolution of structural optimization techniques for steel skeletal structures including industrial design applications. Eng Optim. 2025;57(1):69–129. 10.1080/0305215X.2024.2390130.

[19] Luh GC, Lin CY. Optimal design of truss-structures using particle swarm optimization. Comput Struct. 2011;89(23–24):2221–32. 10.1016/j.compstruc.2011.08.013.

[20] Li LJ, Huang ZB, Liu F, Wu QH. A heuristic particle swarm optimizer for optimization of pin connected structures. Comput Struct. 2007;85(7–8):340–9. 10.1016/j.compstruc.2006.11.020.

[21] Li LJ, Huang ZB, Liu F. A heuristic particle swarm optimization method for truss structures with discrete variables. Comput Struct. 2009;87(7–8):435–43. 10.1016/j.compstruc.2009.01.004.

[22] Kaveh A, Talatahari S. Particle swarm optimizer, ant colony strategy and harmony search scheme hybridized for optimization of truss structures. Comput Struct. 2009;87(5–6):267–83. 10.1016/j.compstruc.2009.01.003.

[23] Torzoni M, Manzoni A, Mariani S. Enhancing Bayesian model updating in structural health monitoring via learnable mappings. Eng Comput. 2025. 10.1007/s00366-025-02224-x.

[24] Sahachaisaree S, Sae-ma P, Nanakorn P. Two-dimensional truss topology design by reinforcement learning. Lect Notes Civ Eng. 2020;80:1237–45. 10.1007/978-981-15-5144-4_122.

[25] Hayashi K, Ohsaki M. Reinforcement learning and graph embedding for binary truss topology optimization under stress and displacement constraints. Front Built Environ. 2020;6:514011. 10.3389/fbuil.2020.00059.

[26] Zhu S, Ohsaki M, Hayashi K, Guo X. Machine-specified ground structures for topology optimization of binary trusses using graph embedding policy network. Adv Eng Softw. 2021. 10.1016/j.advengsoft.2021.103032.

[27] Ororbia ME, Warn GP. Design synthesis through a Markov Decision Process and reinforcement learning framework. J Comput Inf Sci Eng. 2022. 10.1115/1.4051598.

[28] Ororbia ME, Warn GP. Design synthesis of structural systems as a Markov Decision Process solved with deep reinforcement learning. J Mech Des. 2023. 10.1115/1.4056693.

[29] Garayalde G, Rosafalco L, Torzoni M, Corigliano A. Mastering truss structure optimization with tree search. J Mech Des. 2025. 10.1115/1.4068300.

[30] Puiutta E, Veith EMSP. Explainable reinforcement learning: a survey. In: Lecture notes in computer science (including subseries lecture notes in artificial intelligence and lecture notes in bioinformatics), vol 12279 LNCS; 2020. pp. 77–95. 10.1007/978-3-030-57321-8_5.

[31] Sutton RS, Barto AG. Reinforcement learning : an introduction, Second Edition. The MIT Press; 2020.

[32] Browne CB, et al. A survey of Monte Carlo tree search methods. IEEE Trans Comput Intell AI Games. 2012. 10.1109/TCIAIG.2012.2186810.

[33] Luo R, Wang Y, Xiao W, Zhao X. AlphaTruss: Monte Carlo tree search for optimal truss layout design. Buildings. 2022;12(5):641. 10.3390/buildings12050641.

[34] Luo R, Wang Y, Liu Z, Xiao W, Zhao X. A reinforcement learning method for layout design of planar and spatial trusses using kernel regression. Appl Sci (Basel). 2022. 10.3390/app12168227.

[35] Garayalde G, Torzoni M, Bruggi M, Corigliano A. Real-time topology optimization via learnable mappings. Int J Numer Methods Eng. 2024;125(15):e7502. 10.1002/nme.7502.

[36] Belytschko T, Liu WK, Moran B. Nonlinear finite elements for continua and structures. Chichester, UK: John Wiley & Sons, Ltd; 2000.

[37] Bellman R. A Markovian decision process. J Math Mech. 1957;6(5):679–84. 10.1512/iumj.1957.6.56038.

[38] Lipson H. Evolutionary synthesis of kinematic mechanisms. Artif Intell Eng Des Anal Manuf. 2008;22(3):195–205. 10.1017/S0890060408000139.

[39] Kocsis L, Szepesvári C. Bandit based Monte-Carlo planning. In: Lecture notes in computer science (including subseries lecture notes in artificial intelligence and lecture notes in bioinformatics), vol 4212 LNAI; 2006. pp. 282–93. 10.1007/11871842_29.

[40] Ariyurek S, Betin-Can A, Surer E. Enhancing the Monte Carlo tree search algorithm for video game testing. In: IEEE conference on computational intelligence and games, CIG, vol. 2020-August; 2020. pp. 25–32. 10.1109/COG47356.2020.9231670.

[41] Auer P, Fischer P, Cesa-Bianchi N. Finite-time analysis of the multiarmed bandit problem. Mach Learn. 2002;47:235–56. 10.1023/A:1013689704352.

[42] Margolis GB. Hierarchical Monte Carlo tree search for tethered AUV planning. Massachusetts Institute of Technology (MIT).

[43] Rizzieri G, Ferrara L, Cremonesi M. Numerical simulation of the extrusion and layer deposition processes in 3D concrete printing with the particle finite element method. Comput Mech. 2024;73(2):277–95. 10.1007/S00466-023-02367-Y/FIGURES/15.

[44] Sørensen K-JI. From waste to structure: a deep reinforcement learning approach to circular design. Massachusetts Institute of Technology; 2024.

